# Mesoporous Surface-Sulfurized Fe–Co_3_O_4_ Nanosheets Integrated with N/S Co-Doped Graphene as a Robust Bifunctional Electrocatalyst for Oxygen Evolution and Reduction Reactions

**DOI:** 10.3390/molecules28052221

**Published:** 2023-02-27

**Authors:** Lingxue Meng, Yige Wang, Wenwei Liu, Chunlei Fan, Haoxiong Nan, Jiang Wang, Jia Yu

**Affiliations:** 1 School of Science, Hainan University, Haikou 570228, China; 2 Materials Genome Institute, Shanghai University, Shanghai 200444, China; 3 The Key Laboratory of Fuel Cell Technology of Guangdong Province, School of Chemistry and Chemical Engineering, South China University of Technology, Guangzhou 510641, China; 4 Key Laboratory of Advanced Energy Materials Chemistry (Ministry of Education), College of Chemistry, Nankai University, Tianjin 300071, China

**Keywords:** iron-cobalt bimetallic oxides, surface vulcanisation, N/S co-doped graphene, oxygen evolution reaction, oxygen reduction reaction, bifunctional electrocatalyst

## Abstract

Playing a significant role in electrochemical energy conversion and storage systems, heteroatom-doped transition metal oxides are key materials for oxygen-involving reactions. Herein, mesoporous surface-sulfurized Fe–Co_3_O_4_ nanosheets integrated with N/S co-doped graphene (Fe–Co_3_O_4_–S/NSG) were designed as composite bifunctional electrocatalysts for the oxygen evolution reaction (OER) and the oxygen reduction reaction (ORR). Compared with the Co_3_O_4_–S/NSG catalyst, it exhibited superior activity in the alkaline electrolytes by delivering an OER overpotential of 289 mV at 10 mA cm^−2^ and an ORR half-wave potential of 0.77 V vs. RHE. Additionally, Fe–Co_3_O_4_–S/NSG kept stable at 4.2 mA cm^−2^ for 12 h without significant attenuation to render robust durability. This work not only demonstrates the satisfactory effect of the transition-metal cationic modification represented by iron doping on the electrocatalytic performance of Co_3_O_4_, but it also provides a new insight on the design of OER/ORR bifunctional electrocatalysts for efficient energy conversion.

## 1. Introduction

Driven by the urgent demand for renewable energies, developing electrochemical energy conversion and storage systems has become a worldwide priority recently, e.g., metal-air batteries, water-splitting systems, and fuel cells, which possess superior environmental friendliness, and high energy efficiencies for the conversion between chemical energies and electric energies [1,2,3,4]. Among them, the oxygen evolution reaction (OER) and the oxygen reduction reaction (ORR) processes play a significant role, especially in the charge/discharge of rechargeable zinc-air batteries. Nevertheless, due to the complex multi-electron and proton transfer processes, OER/ORR is usually faced with sluggish reaction kinetics which seriously hinders the operation of electrochemical energy conversion and storage systems [5,6]. The precious metals Ru- and Ir-based materials are the most active commercial electrocatalysts for OER, while Pt is the most active ORR electrocatalyst [7,8]. Nonetheless, the scarce storage, high cost, and insufficient stability caused by the aggregation trends greatly impede the large-scale applications of these precious metal-based electrocatalysts. Therefore, it is necessary to develop cheaper alternatives to achieve efficient and durable OER/ORR bifunctional electrocatalytic kinetics. 

Transition metal-based compounds with different crystal structures and variable metal valence states (e.g., the spinel oxides [9,10,11] and the perovskite oxides [12,13]) have intriguing versatility to accelerate the multi-electron transfer in OER/ORR processes. Additionally, their low cost and abundant availability also make them promising as a replacement for precious metal-based catalysts. Among them, the spinel oxides represented by Co_3_O_4_ with a mixed valence of Co^2+^ and Co^3+^ have a series of advantages including the ease of preparation, versatile morphology, and high stability, which are highly desired for electrocatalyst designs, but their poor electrical conductivity and limited active sites limit the full play of intrinsic activity [14,15]. Various strategies including hetero-anion (N, P, S) doping [16], polymetallic complexation [17], oxygen vacancy fabrication [18], and interfacial modulation [19] have been developed to improve the OER/ORR activity of transition metal-based electrocatalysts. For example, Shi et al. designed an N-doped CoS_2_ electrocatalyst, in which CoS_2_ could fast-electron-transfer and which was an excellent OER electrocatalyst in an alkaline environment [20]. DFT studies have shown that N doping can change the electron density of the Co atom and reduce the reaction barrier in the OER process. At the same time, N has a high positive charge density, which can be a catalytically active site for the OER. Thus, the OER overpotential containing N–CoS_2_ at 10 mA cm^−2^ is 240 mV, making it an excellent OER electrocatalyst [21]. Yuan et al. doped S into CoFe phosphide nanoparticles dispersed on N, P, S triple-doped graphene (NPSG), to realize charge redistribution in FeCo_3_P and change the electronic structure around FeCoP [22]. An excellent bifunctional activity in alkaline electrolytes was achieved with an OER overpotential at 10 mA cm^−2^ (*E*_10_) of 290 mV and an ORR half-wave potential (*E*_1/2_) of 0.83 V vs. RHE, which verified the feasibility of anionic modification in improving transition metal-based electrocatalysts. Meanwhile, heteroatom-doped graphene can not only enhance conduction electronic conduction but can also provide a portion of the ORR/OER active sites [23,24]. Besides the well-proved anionic modification, it is interesting and necessary to investigate the influence of the transition-metal cationic modification on electrocatalytic performance.

Herein, an efficient and stable electrocatalyst (Fe–Co_3_O_4_–S/NSG) composed of mesoporous surface-sulfurized iron-cobalt bimetallic oxides nanosheets (Fe–Co_3_O_4_–S) integrated with N/S co-doped graphene (NSG) was synthesized via a successive process including a hydrothermal reaction, a calcination process, and heteroatom doping. When compared with un-Fe-doped Co_3_O_4_–S fabricated under the same conditions, Fe–Co_3_O_4_–S possessed a more uniform structure with superior catalytic activity and stability. Moreover, the N/S co-doped graphene in this composite system contributed to the high dispersion of the transition metal matrix, enlarged the specific surface area, increased the active sites, and thus facilitated the charge transfer. As expected, Fe–Co_3_O_4_–S/NSG provided better OER/ORR bifunctional electrocatalytic activity compared to Co_3_O_4_–S/NSG, with an *E*_10_ decreasing from 322 mV to 289 mV and an *E*_1/2_ increasing from 0.75 V to 0.77 V vs. RHE. Additionally, it maintained its current density for at least 12 h to render considerable stability. This demonstrates the effect of iron and sulfur doping into Co_3_O_4_ to achieve a robust OER/ORR electrocatalyst. 

## 2. Results and Discussion

**Figure 1:**
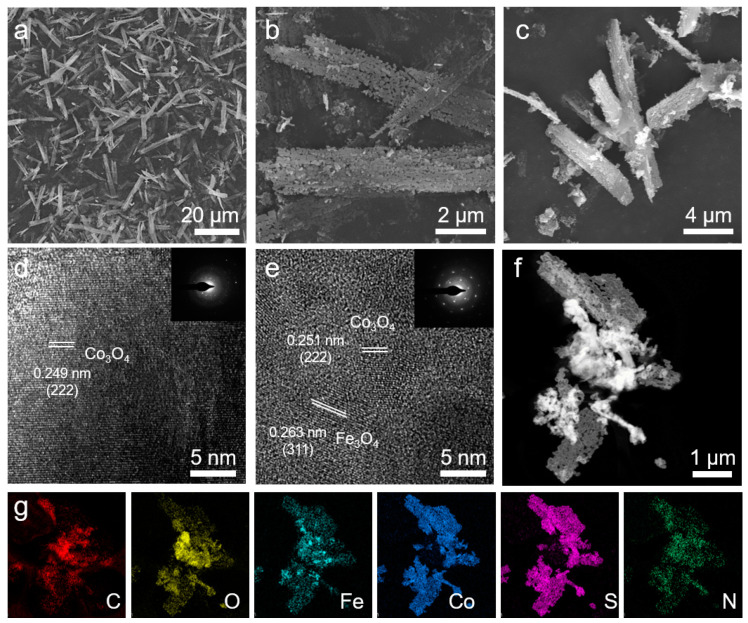
SEM images of (**a**) precursor, (**b**) Fe–Co_3_O_4_, and (**c**) Fe–Co_3_O_4_–S/NSG. HRTEM image of (**d**) Co_3_O_4_–S/NSG and (**e**) Fe–Co_3_O_4_–S/NSG (inset: SAED patterns). (**f**) HAADF-STEM of Fe–Co_3_O_4_–S/NSG image and (**g**) EDS elemental mapping on C, O, Fe, Co, S, and N.

The morphological changes of Fe–Co_3_O_4_–S/NSG during the experiment were recorded by the SEM. The precursors presented a homogeneous striped nanosheet structure with smooth and flat surfaces (Figure 1a). After calcination, abundant pores could be observed from the SEM image of Fe–Co_3_O_4_ (Figure 1b). This structure was attributed to the high temperature during the calcination process, which can increase the specific surface area of the material, facilitate electron transfer, and thus enhance the catalyst performance. The high-temperature calcination did not affect the underlying morphology of the material. The final integration with NSG to form the hybrid catalyst revealed that NSG was adsorbed onto the surface of Fe–Co_3_O_4_–S (Figure 1c).

Subsequently, the microstructures of Co_3_O_4_–S/NSG and Fe–Co_3_O_4_–S/NSG by the TEM were investigated. The lattice fringe of Co_3_O_4_–S/NSG with a spacing of 0.249 nm was attributed to the (222) planes of cubic Co_3_O_4_ (Figure 1d). After the incorporation of the Fe element, the lattice fringe (0.251 nm) of Fe–Co_3_O_4_–S/NSG was consistent with the (222) plane of Co_3_O_4_, which was higher than that of Co_3_O_4_–S/NSG (Figure 1e). This result proves that the Fe element can modulate the configuration of Co_3_O_4_. The lattice edge of Fe–Co_3_O_4_–S/NSG (0.263 nm) is assigned to the (311) plane of Fe_3_O_4_. In addition, porous structures were also observed in the high-angle annular dark field-STEM (HAADF-STEM) image (Figure 1f), which corresponded to the previous SEM results. Elemental mapping images of selected areas of the Fe–Co_3_O_4_–S/NSG samples demonstrated a uniform distribution of C, O, Fe, Co, S, and N, indicating that the S and NSG were successfully adsorbed on the material surface (Figure 1g).

**Figure 2:**
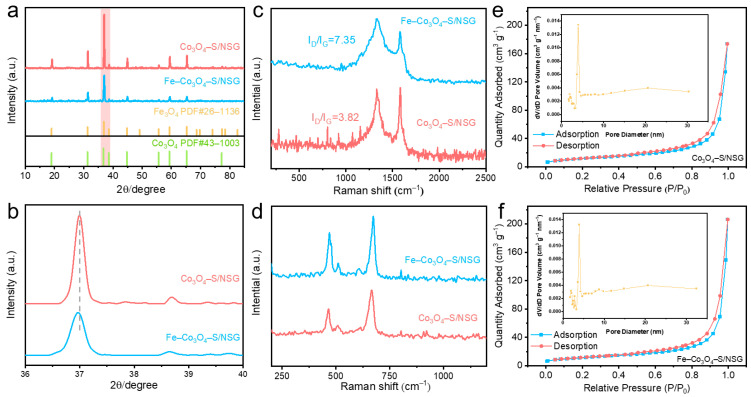
(**a**) XRD patterns of Co_3_O_4_–S/NSG and Fe–Co_3_O_4_–S/NSG along with the JCPDS card of Fe_3_O_4_ and Co_3_O_4_ and (**b**) enlargement of the (311) peak region. (**c**,**d**) Raman spectra and (**e**,**f**) N_2_ adsorption–desorption isotherms (inset: pore size distribution) of Fe–Co_3_O_4_–S/NSG and Co_3_O_4_–S/NSG.

The XRD patterns demonstrated the crystal phase of Co_3_O_4_–S/NSG and Fe–Co_3_O_4_–S/NSG (Figure 2a). The diffraction peaks of Fe–Co_3_O_4_–S/NSG at 19.18°, 31.44°, 36.98°, 44.98°, 59.50°, and 65.4° matched with the planes of the Co_3_O_4_ (PDF#43–1003 [25,26]) and Fe_3_O_4_ (PDF#26–1136 [27,28]), indicating that the synthesized catalyst was a mixture of Co_3_O_4_ and Fe_3_O_4_, and the surface sulphuration did not alter its crystal structure. It is noteworthy that the additional characteristic peaks of Fe_3_O_4_ were not observed, which could be attributed to the small amount of iron doping. Additionally, the peak intensity of Fe–Co_3_O_4_–S/NSG was lower than that of Co_3_O_4_–S/NSG due to the decrease in the proportion of the Co element. The magnified XRD data showed the (311) peaks of 37.02° and 36.98° for Co_3_O_4_–S/NSG and Fe–Co_3_O_4_–S/NSG, respectively, indicating that the incorporation of iron shifted the lattice negatively, causing the lattice spacing to become larger [29] (Figure 2b). These results were consistent with the results of the HRTEM images. The Raman spectra of Fe–Co_3_O_4_–S/NSG and Co_3_O_4_–S/NSG demonstrated a typical D band (sp^3^ hybridized carbon) at 1326.1 cm^−1^ and a G band (sp^2^ graphitic carbon) at 1582.6 cm^−1^ (Figure 2c). The intensity ratios of the D band and the G band (I_D_/I_G_) were calculated to obtain the degree of graphitization of the electrocatalysts [30]. The I_D_/I_G_ values of Co_3_O_4_–S/NSG and Fe–Co_3_O_4_–S/NSG were 3.82 and 7.35, respectively, illustrating a high degree of graphitization of Fe–Co_3_O_4_–S/NSG due to the large structural defects and disordered properties of the synthesized NSG. The Raman peaks of 470.7, 509.4, and 674.4 cm^−1^ could be ascribed to the stretching vibration of Co–O, indicating the presence of the Co–O bond (Figure 2d).

The BET specific surface area (SSA) analysis was performed by the nitrogen adsorption/desorption method. The isotherms and pore size distributions of Co_3_O_4_–S/NSG and Fe–Co_3_O_4_–S/NSG are shown in Figure 2e,f. Both samples had typical IV isotherms with the characteristic of weak adsorbent-adsorbent interactions [31,32]. Specifically, Co_3_O_4_–S/NSG possessed an SSA of 41.82 m^2^ g^−1^ and a pore volume of 0.27 cm^3^ g^−1^. When the material was doped with iron, the SSA was 40.81 m^2^ g^−1^, which was almost the same as the Co_3_O_4_–S/NSG results, indicating little change in the SSA. Ho the pore volume increased to 0.39 cm^3^ g^−1^. The results prove that the incorporation of iron can increase the number of porous structures in the material. Additionally, the inset reveals that the size of the pores was mainly distributed in the range of 2–10 nm. The mesoporous structure can provide abundant channels and highly electrochemically active surfaces, facilitating rapid mass transfer in OER/ORR reactions [33].

**Figure 3:**
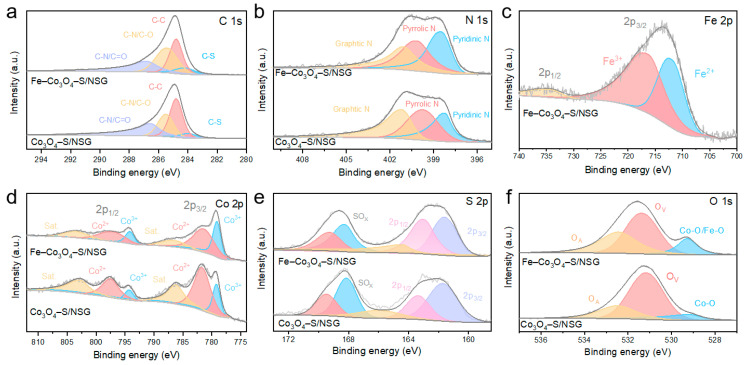
(**a**) C 1s and (**b**) N 1s XPS spectra of Fe–Co_3_O_4_–S/NSG and Co_3_O_4_–S/NSG. (**c**) Fe 2p XPS spectra of Fe–Co_3_O_4_–S/NSG. (**d**) Co 2p (**e**) S 2p and (**f**) O 1s XPS spectra of Fe–Co_3_O_4_–S/NSG and Co_3_O_4_–S/NSG.

XPS spectra were obtained to determine the elemental composition of the catalysts’ surface and the electronic state of these elements. The C 1s spectrum of Fe–Co_3_O_4_–S/NSG showed four peaks at 284.15, 284.80, 285.44, and 286.81 eV, which are the characteristics of the C–S bond, C–C bond, C–N/C–O bond, and C–N/C=O bond, respectively (Figure 3a). The data proves that the nitrogen/sulphur co-doped graphene (NSG) was successfully synthesized. In the high-resolution N 1s spectrum of Fe–Co_3_O_4_–S/NSG, peaks at 398.28, 399.78, and 401.28 eV confirmed the existence of the pyridine N, pyrrole N, and graphite N, respectively (Figure 3b). A previous study has shown that a high pyridine N content is beneficial to the ORR process [34]. Therefore, compared with Fe–Co_3_O_4_–S/NSG, Co_3_O_4_–S/NSG with a slightly higher pyrrole N content possesses a better ORR starting potential, but the difference is not significant. The Fe 2p spectrum of Fe–Co_3_O_4_–S/NSG could be subdivided into two peaks at 717.28 and 713.78 eV, confirming the existence of Fe^3+^ and Fe^2+^, which aligned with the XRD patterns (Figure 3c). In the Co 2p spectra, the absorption peaks of the materials at 781.58/797.58 eV and 779.18/794.18 eV proved the presence of Co^2+^ and Co^3+^ (Figure 3d). Two satellite peaks could be discerned at 786.98 eV and 803.38 eV. Significantly, the peak intensity of Co^2+^ decreased while the peak intensity of Co^3+^ increased after the incorporation of iron, showing that the oxidizing agent Fe^3+^ could convert Co^2+^ into Co^3+^ partially. The increase in the content of Co^3+^ can raise the ratio of Co^3+^ to Co^2+^ in the catalyst, which is conducive to the catalytic reaction [35].

The spin-orbit transitions of S 2p_1/2_ and S 2p_3/2_ concentrated respectively at a binding energy of 163.08 eV and 161.58 eV in Fe–Co_3_O_4_–S/NSG (Figure 3e). Moreover, the peaks centred at 164.58 eV, 168.08 eV, and 169.28 eV were indexed to the characteristic peaks of sulphur oxides, which are located by surface oxidation during vulcanization. The S 2p_3/2_ orbital area of Fe–Co_3_O_4_–S/NSG was significantly higher than that of Co_3_O_4_–S/NSG, while the peak intensity of the sulphur oxides decreased after the doping of iron. This result indicates that the incorporation of iron can make sulphur atoms combine with Fe ions into the interior of the material, resulting in the decrease of the sulphur content on the surface of the material. Since transition metal sulfides have greater catalytic activity than transition metal oxides, Fe–Co_3_O_4_–S/NSG demonstrates superior OER/ORR performance. The O 1s spectra disclosed three absorption peaks, located at 532.48 eV for adsorbed oxygen, 531.38 eV for oxygen vacancies, and 529.18 eV for lattice oxygen, respectively (Figure 3f). After the incorporation of iron, the peak area of the typical metal-oxygen bond (M–O) at 529.18 eV increased obviously, which further proves that the iron was successfully doped. Meanwhile, the peak area of adsorbed oxygen also increased significantly because the oxygen in the air filled the surface vacancy caused by sulphur entering the internal position of the catalyst [36]. This result is consistent with that obtained from the high-resolution XPS spectra of S. Additionally, ICP– MS is used to detect the concentration of the elements within the materials. The results display that the proportion of Co and Fe in Fe–Co_3_O_4_–S/NSG is 34.87% and 3.16%, respectively, approximating the synthetic phase.

**Figure 4:**
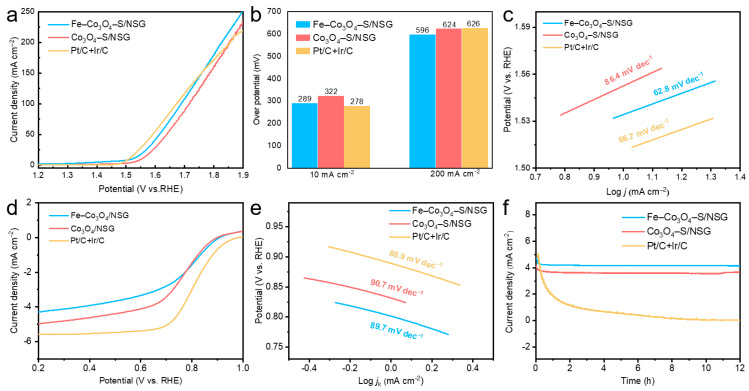
(**a**) OER polarization curves, (**b**) overpotentials at 10 and 200 mA cm^−2^ current densities, (**c**) corresponding Tafel slopes, (**d**) ORR LSV at a rotation speed of 1600 rpm and (**e**) corresponding Tafel slopes of Co_3_O_4_–S/NSG, Fe–Co_3_O_4_–S/NSG and Pt/C + Ir/C electrocatalysts. (**f**) Chronoamperometric responses of Fe–Co_3_O_4_–S/NSG, Co_3_O_4_–S/NSG and commercial Pt/C + Ir/C at 1.524 V.

To evaluate the electrochemical properties of the as-prepared samples, LSV and CV tests were conducted in alkaline electrolytes using a three-electrode system. The mixture of commercial electrocatalysts Pt/C and Ir/C (mass ratio = 1:1) was included for comparison. The OER polarization curves of Fe–Co_3_O_4_–S/NSG, Co_3_O_4_–S/NSG, and Pt/C + Ir/C showed that at low current density, the overpotential of Fe–Co_3_O_4_–S/NSG was just lower than that of Co_3_O_4_–S/NSG. As the current density increased, the overpotential of Fe–Co_3_O_4_–S/NSG was even lower than that of Pt/C + Ir/C catalyst (Figure 4a). When the current density was 10 mA cm^−2^, Fe–Co_3_O_4_–S/NSG exhibited an overpotential of 289 mV, which was lower than that of Co_3_O_4_–S/NSG (322 mV) (Figure 4b). When the current density was 200 mA cm^−2^, Fe–Co_3_O_4_–S/NSG exhibited an overpotential of 594 mV, which was lower than that of Co_3_O_4_–S/NSG (624 mV), and Pt/C + Ir/C (626 mV). The Tafel slope curve of Fe–Co_3_O_4_–S/NSG showed a slope of 62.8 mV dec^−1^, which was better than that of the commercial noble metal Pt/C + Ir/C catalyst (66.7 mV dec^−1^), while Co_3_O_4_–S/NSG delivered the largest slope of 86.4 mV dec^−1^ (Figure 4c). The ORR LSV curve of Fe–Co_3_O_4_–S/NSG gave an *E*_1/2_ of 0.77 V, which was higher than that of Co_3_O_4_–S/NSG (0.75 V) to show superior ORR performance (Figure 4d). Intriguingly, Fe–Co_3_O_4_–S/NSG exhibited a small Tafel slope of 89.7 mV dec^−1^, which outperformed Co_3_O_4_–S/NSG (90.7 mV dec^−1^) and was close to Pt/C + Ir/C (88.9 mV dec^−1^), demonstrating the fast kinetics of ORR on Fe–Co_3_O_4_–S/NSG (Figure 4e). The results confirm that Fe–Co_3_O_4_–S/NSG has excellent ORR/OER activity compared to the un-Fe-doped sample. The robust performance could be attributed to the synergistic effect between the alloy component and the defect-rich carbon carrier, which helps to expose more active sites and accelerate electron transport during the ORR/OER process [37,38,39]. The chronoamperometry (CA) measurement can be employed to test the stability [40,41] of Fe–Co_3_O_4_–S/NSG and Co_3_O_4_–S/NSG at 1.524 V. It was observed that the current densities of Fe–Co_3_O_4_–S/NSG and Co_3_O_4_–S/NSG were stable at 4.2 mA cm^−2^ and 3.6 mA cm^−2^, respectively, without significant attenuation within 12 h. It was found that Fe–Co_3_O_4_–S/NSG and Co_3_O_4_–S/NSG have better electrochemical stability than commercial Pt/C + Ir/C (Figure 4f).

**Figure 5:**
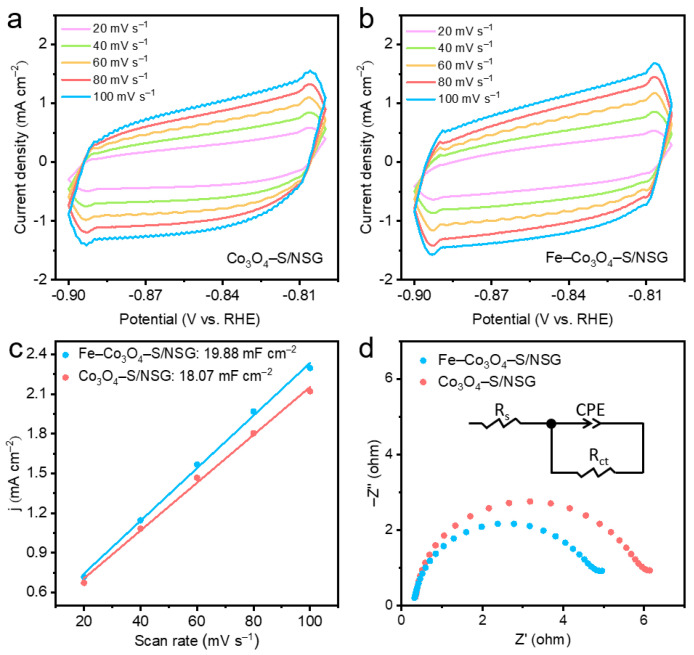
The CV curves at various scan rates of 20, 40, 60, 80, and 100 mV s^−1^ for (**a**) Co_3_O_4_–S/NSG and (**b**) Fe–Co_3_O_4_–S/NSG. (**c**) The corresponding value of *C*_dl_. (**d**) EIS spectra of Co_3_O_4_–S/NSG and Fe–Co_3_O_4_–S/NSG, the illustration shows the corresponding equivalent circuit.

Because of the positive relationship, an electrocatalyst with a higher *C*_dl_ value possesses a larger ECSA, which usually displays better electrocatalytic activity [42,43]. According to the CV curves of Co_3_O_4_–S/NSG and Fe–Co_3_O_4_–S/NSG at different scanning rates (20–100 mV s^−1^), the shapes of the CV curves remained stable while their area changed with the increase in the scanning rate (Figure 5a,b). The *C*_dl_ values for Co_3_O_4_–S/NSG and Fe–Co_3_O_4_–S/NSG were 18.07 and 19.88 mF cm^−2^, respectively (Figure 5c). Combined with the BET results, Fe–Co_3_O_4_–S/NSG possessed a larger ECSA due to the incorporation of iron. The EIS test was carried out at an overpotential of 298 mV, when the semicircular diameter of Fe–Co_3_O_4_–S/NSG was smaller than that of Co_3_O_4_–S/NSG (Figure 5d). The inset shows the corresponding equivalent circuit, where R_s_ indicates the solution resistance, CPE is the constant phase element and R_ct_ is the charge transfer resistance [44,45] After Z-view fitting, the equivalent circuit fit resulted in a measured charge transfer resistance of Fe–Co_3_O_4_–S/NSG measured to be 4.674 Ω, which was lower than that of Co_3_O_4_–S/NSG (5.791 Ω), indicating the fastest reaction kinetics of Fe–Co_3_O_4_–S/NSG. This result should be attributed to the incorporation of iron, which effectively improves charge transfer and facilitates electrocatalytic activity.

## 3. Experimental Section

### 3.1. Material Preparation

All chemicals and materials were analytical grades without further purification. Ethanol (C_2_H_5_OH, AR 99.7%), silicon dioxide (SiO_2_, AR 99%, 12~15 nm), and sulfuric acid (H_2_SO_4_, GR 99.5%) were purchased from Sinopharm Chemical Reagent Co., Ltd., Shanghai, China. Cobalt nitrate hexahydrate (Co(NO_3_)_2_·6H_2_O, AR 99%), iron nitrate nonahydrate (Fe(NO_3_)_3_·9H_2_O, AR 99%), urea (CH_4_N_2_O, AR 99%), ammonia (NH_3_·H_2_O, AR 25~28%), sodium sulfide nonahydrate (Na_2_S·9H_2_O, AR 98%), potassium hydroxide (KOH, GR 95%), and isopropanol (C_3_H_8_O, AR, 99.5%) were obtained from MACKLIN Reagent, Shanghai, China. Graphene powde r (C, AR 99.95%, 80~120 meshes), hydrogen peroxide (H_2_O_2_, AR 30%), potassium permanganate (KMnO_4_, AR 99%), and barium chloride (BaCl_2_, AR 98%) were taken from RHAWN Reagent, Shanghai, China. Sodium nitrate (NaNO_3_, AR 99%), melamine (C_3_H_6_N_6_, AR 99%), dibenzyl disulfide (C_14_H_14_S_2_, AR 99%), and hydrogen fluoride (HF, AR 40%) were purchased from Aladdin, Shanghai, China. The 5 wt. % Na fion solution was obtained from Du-Pont. 20 wt. % Pt/C and 20 wt. % Ir/C were purchased from Suzhou Yilongsheng Energy Technology Co., Ltd., Suzhou, China.

### 3.2. Material Synthesis

**Figure 6:**
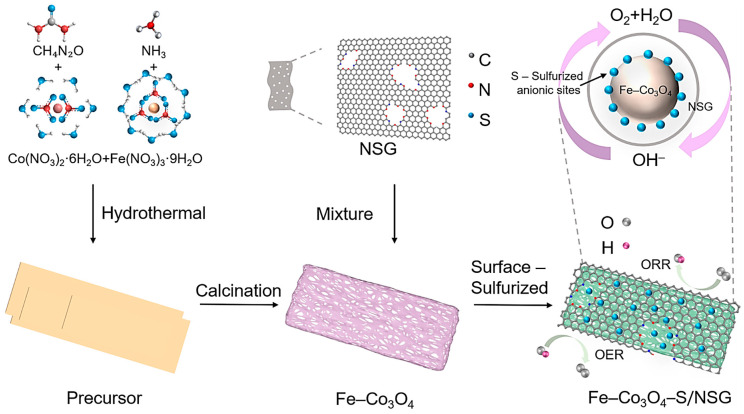
Schematic synthesis route of Fe–Co_3_O_4_–S/NSG as an OER/ORR bifunctional electrocatalyst.

Fe–Co_3_O_4_–S/NSG was fabricated by a three-step process, including hydrothermal synthesis of the precursor nanosheets, calcination to obtain Fe–Co_3_O_4_, and finally integration with NSG, along with surface vulcanisation to obtain the target product (Figure 6). The specific synthesis process is as follows.

#### 3.2.1. Synthesis of Co_3_O_4_ and Fe–Co_3_O_4_

Fe–Co_3_O_4_ was synthesized by hydrothermal method and calcination methods. The specific process was as follows: 4.5 mM Co(NO_3_)_2_·6H_2_O and 0.5 mM Fe(NO_3_)_3_·9H_2_O were dissolved in 35 ml ultrapure water. Subsequently, 10 mM urea and 20 mM ammonia solution were added as precipitants. The obtained solution was stirred using a magnetic stirrer for 20 min and followed by sonication for 5 min. After that, the mixed solution was placed in a PTFE-lined autoclave at 170 °C for 9.5 h. Once cooled down to room temperature, the obtained solution was slowly washed with ethanol and ultrapure water, respectively. After drying, the pink powder was obtained. The synthesis of Co_3_O_4_ was carried out by dissolving 5 mM Co(NO_3_)_2_·6H_2_O in 35 mL of ultrapure water. The remainder of the steps were the same as for the synthesis of Fe–Co_3_O_4_.

#### 3.2.2. Synthesis of NSG 

Graphene oxide (GO) was synthesized by using modified Hummer methods. An amount of 0.1 g GO and 0.5 g hydrophilic SiO_2_ nanoparticles (12–15 nm) were uniformly dispersed in 500 mL ethanol by ultrasonication. The ethanol was then evaporated using a rotary evaporator at 80 °C to obtain a flake of GO/SiO_2_ solid. Subsequently, 0.5 g melamine and 0.5 g dibenzyl disulfide (BDS) were added to the solids and ground into fine powder. The mixture was heated at 900 °C for 1 h in a nitrogen-filled atmosphere with a heating rate of 5 °C min^−1^. After that, nitrogen/sulfur co-doped graphene (NSG/SiO_2_) loaded with silica was synthesized. Next, the obtained NSG/SiO_2_ was placed in hydrofluoric acid (HF) solution for 12 h to remove the silica. The product was then cleaned several times with ultrapure water and ethanol, followed by drying at 70 °C under the condition of oxygen isolation. After drying, NSG was synthesized.

#### 3.2.3. Synthesis of Co_3_O_4_–S/NSG and Fe–Co_3_O_4_–S/NSG 

Fe–Co_3_O_4_–S/NSG or Co_3_O_4_–S/NSG was prepared by ultrasonic mixing. NSG and Fe–Co_3_O_4_ or Co_3_O_4_ were mixed in a mass ratio of 1:4 and sonicated in a 0.4 mol L^−1^ solution of sodium sulfide solution for 2 h and then left to stand for 24 h. Subsequently, the solids were washed and dried at 60 °C for 8 h using a filtration technique to obtain the target product Fe–Co_3_O_4_–S/NSG or Co_3_O_4_–S/NSG electrocatalyst.

### 3.3. Physicochemical Characterizations

The structural and morphological characteristics of the synthetic material were determined with a scanning electron microscope (SEM) (TESCAN MIRA LMS, Brno, Czech Republic) and a scanning transmission electron microscopy (STEM) (FEI Tecnai G2 F20, Hillsboro, OR, USA). The crystal phase analysis of the synthetic catalysts was elucidated by X-ray powder diffraction (XRD) (Rigaku Smartlab 9 kW, using an X-ray diffractometer over the range of 10° to 90° 2θ, Tokyo, Japan). The specific surface areas of the materials were calculated by a Brunauer-Emmett-Teller (BET) (Micromeritics APSP 2460, 77k, Norcross, GA, USA). Adopting the Barrett-Joyner-Halenda (BJH) method, pore size distribution was calculated from the desorption branch of the N_2_ desorption isotherm. The chemical states and composition of materials were tested by X-ray photoelectron spectroscopy (XPS) (Thermo Scientific K–Alpha+ spectrometer, Shanghai, China). All spectra were calibrated using the C 1s peak energy of 284.8 eV binding energy standard peak. The elemental content of materials can be precisely detected by inductively coupled plasma mass spectroscopy (ICP-MS) (Agilent 7700s, Beijing, China). The synthesized samples were qualitatively analysed by using Raman spectra (LabRam HR Evolution, using an Ar-ion laser beam λ = 514 nm Shanghai, China).

### 3.4. Electrochemical Measurements

Utilizing a three-electrode system, electrochemical measurements were conducted in CHI 760E electrochemical workstation. The instrument was subjected to 95% iR compensation before the test. Taking 1 mol L^−1^ potassium hydroxide solution in an oxygen atmosphere as the electrolyte in the OER tests, a rotating disc-shaped glassy carbon electrode (RDE) with a diameter of 5 mm and an area of 0.19625 cm^2^ was employed as the working electrode, a carbon rod was utilized as the counter electrode, and a Hg/HgO electrode was used as the reference electrode. During the test, the RDE was coated with different catalyst slurries. A high-speed rotator (Pine Instruments) was used in the ORR tests, the RDE coated with catalyst ink was utilized as the working electrode, a platinum wire was used as the counter electrode, and a Hg/HgO electrode was employed as the reference electrode. The electrolyte was 0.1 mol L^−1^ KOH solution in an oxygen sufficient atmosphere. The obtained potentials (*E*_Hg/HgO_) were converted to an RHE scale utilizing the following Nernst equation: *E*_RHE_ = *E*_Hg/HgO_ + 0.098 + 0.059 × pH (1 M KOH, pH~14; 0.1 M KOH, PH~13), where *E*_RHE_ represents the reversible potential and *E*_Hg/HgO_ is the potential measured against the reference electrode. 

To prepare the catalyst ink, 5 mg of Co_3_O_4_–S/NSG and 5 mg of Fe–Co_3_O_4_–S/NSG were dispersed in a solution containing 570 μL of isopropanol, 570 μL of anhydrous ethanol, 285 μL of ultrapure water, and 75 μL of 5 wt% Nafion, respectively. The suspension was then sonicated until it became a homogeneous ink-like consistency. An amount of 20 μL of the prepared catalyst was taken and added dropwise to the working electrode and 2.5 mg Pt/C and 2.5 mg Ir/C were dispersed in a solution containing 1425 μL of anhydrous ethanol and 75 μL of 5 wt% Nafion, which was then sonicated until it became homogeneous and ink-like. Subsequently, an amount of 5 μL of the prepared noble metal catalyst was taken and added dropwise to the working electrode. The Tafel plots were derived from the analysis of the linear sweep voltammetry (LSV) test with a scanning rate of 5 mV s^−1^. The Tafel slopes were calculated by the formula *η* = a ± b log *|j|*, where *η* is the overpotential, *j* represents the current density, a is the overpotential at a current density of 1 mA cm^−2^, and b represents the Tafel slope. Additionally, electrochemical impedance spectroscopy (EIS) tests were used to investigate the properties of materials and electrode reactions in the frequency range of 0.1 Hz to 100 kHz. Since the electrical double-layer capacitor (*C*_dl_) is proportional to the electrochemically active surface area (ECSA), cyclic voltammetry (CV) measurements were conducted to study the reaction mechanisms within the static non-faradaic region. The CV tests were carried out between −0.9 and −0.8 V (vs. SCE) at the different scan rates of 20, 40, 60, 80, and 100 mV s^−1^, respectively. The *C*_dl_ was calculated by selecting the current density difference at a potential of –0.85 V at different rates and fitting the current density difference value to the sweep speed linearly. Half of the slope of the fitted straight line was the *C*_dl_. The chrono-current measurement was adopted to access the stability of the materials at 1.524 V for 12 h.

## 4. Conclusions

An efficient and durable OER/ORR bifunctional electrocatalyst (Fe–Co_3_O_4_–S/NSG) was developed by integrating sulfur/iron co-doped Co_3_O_4_ (Fe–Co_3_O_4_–S) and nitrogen/sulfur co-doped graphene (NSG). The resulting material, Fe–Co_3_O_4_–S/NSG, had a homogeneous porous nanosheet structure, which could increase the number of active sites and accelerate electron transfer. Furthermore, the addition of NSG further increased the specific surface area of the material and improved the conductivity of the catalyst. In terms of performance, the Fe–Co_3_O_4_–S/NSG electrocatalyst demonstrated excellent bifunctional activity with an OER *E*_10_ of 289 mV and an ORR *E*_1/2_ of 0.77 V vs. RHE. Additionally, it could be stabilized at 4.2 mA cm^−2^ for 12 h, which is superior to the commercial Pt/C + Ir/C electrocatalyst. This work demonstrates that sulphated Iron-cobalt bimetallic oxides combined with nitrogen and sulfuric doped graphene can improve the overall OER and ORR performance of the catalyst. This provides a new approach to the design of low-cost and high-performance electrocatalysts for use in energy conversion and storage.

## Data Availability

Not applicable.

## Sample Availability

Samples of the compound Fe–Co_3_O_4_–S/NSG is available from the authors.
